# Prediction of Nodal Involvement in Primary Rectal Carcinoma without Invasion to Pelvic Structures: Accuracy of Preoperative CT, MR, and DWIBS Assessments Relative to Histopathologic Findings

**DOI:** 10.1371/journal.pone.0092779

**Published:** 2014-04-02

**Authors:** Jun Zhou, Songhua Zhan, Qiong Zhu, Hangjun Gong, Yidong Wang, Desheng Fan, Zhigang Gong, Yanwen Huang

**Affiliations:** 1 Department of Radiology, Shuguang Hospital, Shanghai University of Traditional Chinese Medicine, Shanghai, China; 2 Department of General Surgery, Shuguang Hospital, Shanghai University of Traditional Chinese Medicine, Shanghai, China; 3 Department of Pathology, Shuguang Hospital, Shanghai University of Traditional Chinese Medicine, Shanghai, China; Istituto dei tumori Fondazione Pascale, Italy

## Abstract

**Objective:**

To investigate the accuracy of preoperative computed tomography (CT), magnetic resonance (MR) imaging and diffusion-weighted imaging with background body signal suppression (DWIBS) in the prediction of nodal involvement in primary rectal carcinoma patients in the absence of tumor invasion into pelvic structures.

**Methods and Materials:**

Fifty-two subjects with primary rectal cancer were preoperatively assessed by CT and MRI at 1.5 T with a phased-array coil. Preoperative lymph node staging with imaging modalities (CT, MRI, and DWIBS) were compared with the final histological findings.

**Results:**

The accuracy of CT, MRI, and DWIBS were 57.7%, 63.5%, and 40.4%. The accuracy of DWIBS with higher sensitivity and negative predictive value for evaluating primary rectal cancer patients was lower than that of CT and MRI. Nodal staging agreement between imaging and pathology was fairly strong for CT and MRI (Kappa value = 0.331 and 0.348, P<0.01) but was relatively weaker for DWIBS (Kappa value = 0.174, P<0.05). The accuracy was 57.7% and 59.6%, respectively, for CT and MRI when the lymph node border information was used as the criteria, and was 57.7% and 61.5%, respectively, for enhanced CT and MRI when the lymph node enhancement pattern was used as the criteria.

**Conclusion:**

MRI is more accurate than CT in predicting nodal involvement in primary rectal carcinoma patients in the absence of tumor invasion into pelvic structures. DWIBS has a great diagnostic value in differentiating small malignant from benign lymph nodes.

## Introduction

Rectal cancer is a common disease and a major cause of mortality in Western countries [1]–[3], in recent years, the prevalence of rectal cancer in China is rapidly increasing, largely due to changes in lifestyle [4]. Prediction of nodal staging in patients with primary rectal carcinoma is important for prognosis, and preoperative assessment of lymph node involvement has important value in developing the therapeutic schedule and a new auxiliary treatment [5]–[8].

Multi-detector-row CT (MDCT) can provide high-quality thin section CT volume images and high resolution reconstructed images in a short examination time in the routine clinical applications [9], [10]. The use of new software has significantly reduced the demand for radiation dose [11]. CT image can provide comprehensive information on spatial relationships among tumors, lymph nodes, and vicinity structures of the rectum. Enhanced CT [8] is currently widely used for the detection metastasis of the peritoneal and pelvic cavity, especially in patients with rich intraperitoneal fat tissue, but enhanced CT is not very efficient in discriminating enlarged lymph node from blood vessels.

MR can effectively detect the lymphatic metastasis due to its good contrast resolution [1], [8], [12]–[14]. Previous studies on the prediction of lymph node involvement are mainly conducted based on lymph node size, morphology and the specific contrast agent [5], [12], [15], [16]. Although prediction using the size criteria is one of the most efficient and relatively effective methods, to date there has been no consensus on the size threshold. Some authors suggested that as long as any lymph node is present in the mesorectum positive lymph node involvement should be considered [12], while some others use 3 mm to 10 mm as threshold value of lymph node involvement [12], [15], [16]. In this study, 4 mm (long-axis of nodes) was taken as a threshold value for nodal positivity. There are studies showing that border contour and signal intensity characteristics of a lymph node can be used as more effective indicators of prognosis than lymph node size [12]. Conventional enhanced MRI can produce good quality images with high resolutions and a relatively large field of view; however, small lymph nodes are easy to be interfered by small blood vessels adjacent to the colorectum, thus generating confusing results.

Currently, with significant progress made in the development of MR contrast agents, numerous MR-specific contrast agents have been identified. Among these, ultra-small superparamagnetic iron oxide (USPIO) holds great promise in the detection of lymph node metastases [5], [17]. Previously reported results have shown that superparamagnetic iron oxide has a very high sensitivity and specificity in the detection of lymph node metastases [5], but it has not been widely used in clinics.

Diffusion-weighted magnetic resonance imaging (DWI), a new MRI technique, is capable of providing functional information of solid tissues and thus considered useful in the detection and characterization of malignant tumors [18], including primary rectal cancer [19], [20]. Takahara et al. [21] reported a unique concept of DWIBS. This technique intentionally uses free breathing scanning rather than breathholding or respiratory triggering to visualize (moving) visceral organs and their lesions. DWIBS, the only noninvasive imaging method, has been gradually applied in the examination of tumor function with the advent of rapid-sequence MR scanning [18], [22], [23], rapid-sequence MR scanning can provide intuitive image for the preoperative evaluation of node involvement in primary rectal cancer patients compared with conventional MR [23].

No report has clarified the comparison of CT, MRI and DWIBS regarding the accuracy of preoperative assessments for lymph node imaging. The aim of this study was to evaluate the clinical value of enhanced CT, high-resolution enhanced MR, and DWIBS in predicting nodal involvement in primary rectal carcinoma in the absence of tumor invasion into pelvic structures before mesorectal excision.

## Materials and Methods

### Patients

The study was approved by the ethics committee of Shuguang Hospital, Shanghai University of Traditional Chinese Medicine, and written informed consent was obtained from all subjects. A total of 52 patients (29 males with a mean age of 62±10 years, 23 females with a mean age of 65±10 years) with histologically confirmed primary rectal carcinoma were recruited between March 2010 and May 2013. Inclusion criteria were: 1) a suspected diagnosis of rectal carcinoma following colonoscopy or rectal CT and MR, from which clear images without apparent artifacts were obtained; 2) total mesorectal excision (TME) surgery within two weeks after radiological examinations; and 3) no preoperative chemoradiotherapy or other tumor treatment.

### CT Examination

The region between the iliac chest and the pubic symphysis was scanned with a MDCT scanner (LightSpeed 64; GE Medical Systems, Milwaukee, WI, USA) in the supine position the following parameters: 120 kVp; 250 mA; 64 sections of 5 mm in thickness with a gap of 5 mm and a collimation width of 0.625 mm. Enhanced CT images were obtained after the intravenous administration of iopamidol (Shanghai Bracco Sine Pharmaceutical Corp. Ltd., China) at a dose of 100 mL (300 mg of iodine per milliliter) and a rate of 3 mL/sec.

Plain and enhanced CT scanning was performed in all subjects during the portal venous phase that was determined with bolus tracking and automated triggering technology. Scan delay time in the artery phase and venous phase were 35 ∼ 40 s and 70 ∼ 80 s, respectively. The transverse section data were reconstructed with 0.625-mm-thick sections and then evaluated with PACS.

### MRI Examination

MRI was performed using the 1.5T whole body system (Philips Medical Systems, Achieva and Intera). Abdominal and pelvic examinations consisted of T1-weighted and T2-weighted MRI. All studies were performed with a sixteen-channel body surface coil. No bowel preparation was performed before examination. The patients were placed in the supine position and positioned feet first on the table platform, ensuring that the body was covered from head to thigh. No contrast agents (either positive or negative) or no intestinal peristalsis-reducing drugs were injected. Axial T1-weighted images with fat saturation (turbospin echo; repetition time (TR) 4.6 ms and echo time (TE) 2.2 ms) were obtained with a slice thickness of 1 mm, section gap of 0 mm, NEX of 1, field of view (FOV) of 350 mm, matrix of 380×264, and scan time of about 2 min 45 s. Sagittal and axial T2-weighted images (turbospin echo; TR 2400–3200 ms, TE 100 ms) were obtained with a slice thickness of 3 mm, section gap of 0 mm, NEX of 3, FOV of 350 mm, matrix of 264×208, and scan time of about 2 min 25 s. DWIBS was performed in the axial plane with the following parameters: TR 1500 ms, TE 42 ms, FOV 420 mm, matrix 128×80, 4-mm slice thickness, no section gap, NEX 5–7, b value 500 s/mm^2^. When enhanced MR was needed, gadopentetate dimeglumine (BeiLu pharmaceutical co, Beijing) was injected into the elbow vein at a dose of 15 ml and a ratio of 1 mL/sec by high-pressure syringe. The contrast agent to patient weight ratio used was 0. 2 ml/kg.

### Image Analysis

All acquired images were analyzed on the PACS workstation (Neusoft, Shenyang, China) prior to surgery and before the pathologic results were known. Two abdominal radiologists with more than 20 years of experience in abdominal and pelvic cavity MR who were blind to the clinical and histopathological information interpreted each of the MR images independently. Differences in assessment were resolved by means of consensus. Images were magnified two to three times for optimal visualization of the mesorectal nodes and were assessed for nodal size, patterns of nodal enhancement, and nodal signal intensity ratio. DWIBS can be acquired by PACS which can change DWI into DWIBS through reverse function. Lymph node positioning was determined by DWIBS scan image combined with T1-weighted and T2-weighted MR images.

#### Nodal metastasis

Lymph node metastasis was determined on the basis of irregular border or mixed signal intensity characteristic of lymph nodes revealed on the CT and MR images and of hyperintense signals of lymph nodes on the DWIBS images.

### Image Outcome Evaluation

#### Nodal size

The maximum bidimensional length in the orthogonal plane of each mesorectal node was measured to the nearest millimeter on the transverse images with the caliper tool on the workstation. Each node was numbered and annotated accordingly on the image.

Lymph node border contour, signal intensity and morphology were also assessed. The borders for each node were classified as either “smooth and well-defined” or “irregular and ill-defined”. The signal intensity within a given node was graded as hypointense, isointense, or hyperintense relative to the signal of the primary tumor. The information regarding whether the signal within the node was uniform, homogeneous or mixed with foci of different intensities was also recorded. The pattern of enhancement was categorized as heterogeneous (peripheral rim-like) or homogeneous. The node was characterized based on the agreement between the two independent observers.

### Histopathologic Evaluation

CT and MR images of preoperative specimen were comparatively analyzed with histopathologic findings. Prior to tissue sectioning, the specimens were photographed. After total mesorectal excision, all visible resected specimens were embedded in paraffin for 24 hours and examined histologically with hematoxylin-eosin (HE) staining. Tumor staging was performed using the TNM (tumor, node and metastasis) system. The extent of local tumor spread in each slice was assessed according to the tumor component of the TNM system. The total number of lymph node was recorded and nodal size including the long-axis was measured in mm. The lymph node status was graded as previously described [2]: N0, no lymph node metastasis; N1, one to three lymph node metastasis; N2, four or more lymph node metastasis.

### Statistical Analysis

Analyses were performed by the SPSS software, version 13.0 (SPSS, Chicago, IL, USA). Accuracy, sensitivity, specificity, positive predictive value (PPV), and negative predictive value (NPV) were calculated for N stage based on CT, MRI, and DWIBS images. Preoperative assessment results from these three methods were compared with the pathologic findings on the Kappa value, which was categorized as previously described [24] as: slight agreement (0.01∼0.20), fair agreement (0.21∼0.40), moderate agreement (0.41∼0.60), substantial agreement (0.61∼0.80), and almost perfect agreement 0.81∼0.99. Differences were considered statistically significant when P<0.05.

## Results

### Highly Matched CT and MR Results with Histological Findings

A total of 635 lymph nodes were obtained from 52 patients, with 6 to 28 (mean: 12.2±4.1) from each patient. The long-axis of the nodes ranged from 1 to 20 mm (mean: 4.4±2.3) and was less than 4 mm in 235 (37%) of the 635 specimens. One hundred and eleven (17.5%) lymph nodes were malignant, of which 45 were in the middle and upper rectum (N0 stage 16, N1 stage 5, N2 stage 6), 35 in the middle rectum (N0 stage 7, N1 stage 1, N2 stage 5), and 31 in the middle and lower rectum (N0 stage 6, N1 stage 3, N2 stage 3). CT and MR results were matched perfectly with the final histological findings in 289 lymph nodes. According to the histopathological analysis, 23 out of 52 patients were at N1 and N2 stages and 29 patients at N0 stage.

### Sensitivity, Specificity, Positive Predictive Value, Negative Predictive Value, and Overall Accuracy in 3 Modalities

When compared with the histological results, the sensitivity, specificity, positive predictive value, negative predictive value, and overall accuracy were 78.3, 65.5, 64.3, 79.2, and 57.7%, respectively, for the CT assessment; 56.5, 82.8, 72.2, 70.6, 63.5%, respectively, for the MRI assessment, and 100, 65.5, 69.7, 100, 40.4%, respectively, for the DWIBS assessment. MRI was superior to CT in terms of specificity, positive predictive value, and accuracy. DWIBS showed higher sensitivity and negative predictive value than CT and MRI (Table 1).

**Table 1:** The accuracy of preoperative nodal staging at CT, MR, and DWIBS (cases, n = 52).

Methods	Sensitivity	Specificity	Positive predictive value	Negative predictive value	Accuracy
CT	18/23 (78.3%)	19/29 (65.5%)	18/28 (64.3%)	19/24 (79.2%)	30/52 (57.7%)
MRI	13/23 (56.5%)	24/29 (82.8%)	13/18 (72.2%)	24/34 (70.6%)	33/52 (63.5%)
DWIBS	23/23 (100%)	19/29 (65.5%)	23/33 (69.7%)	7/7 (100%)	21/52 (40.4%)

### Nodal Staging Data of CT, MRI and DWIBS are Agreement with Pathologic Results

**Figure 1:**
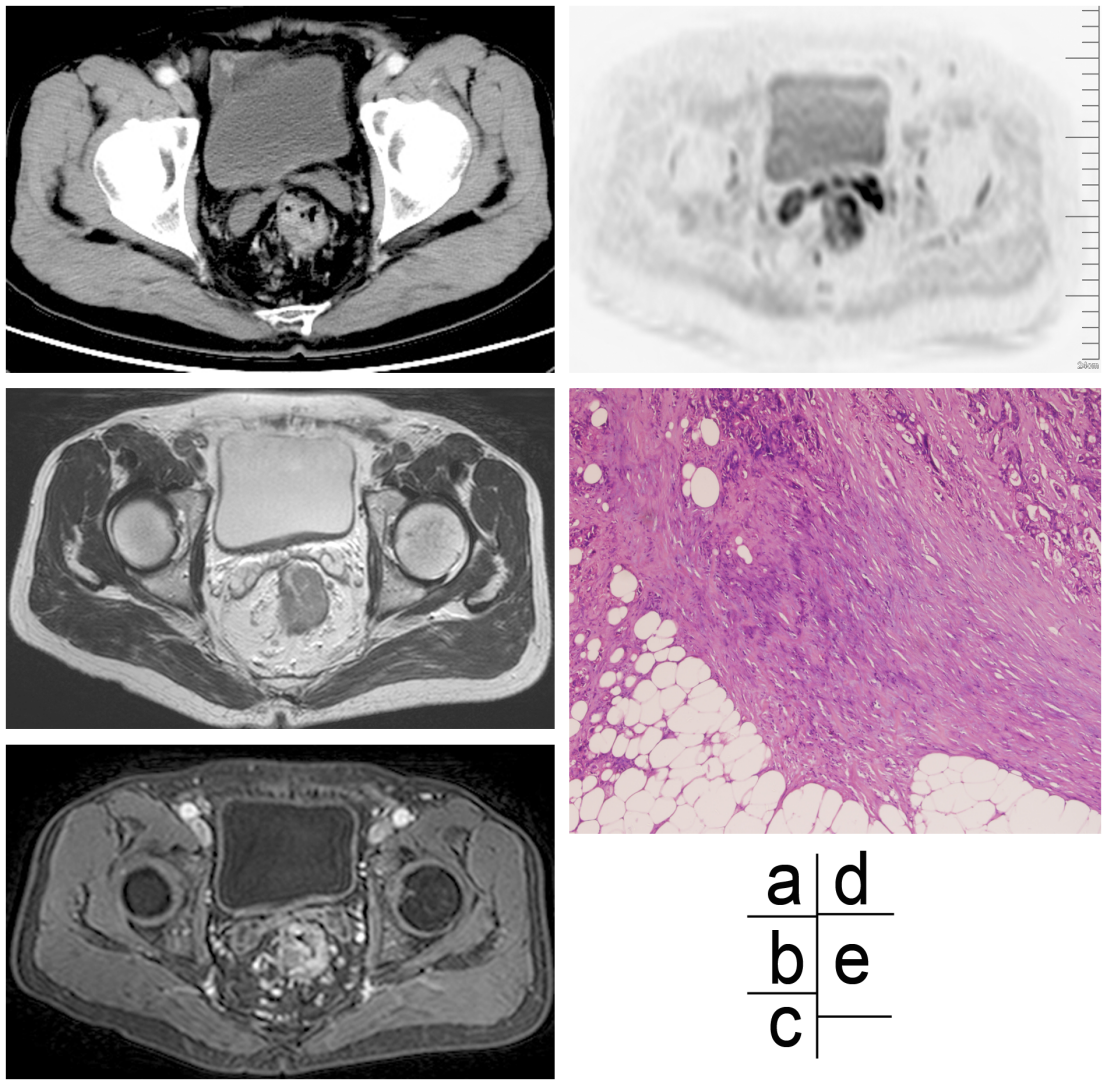
Imaging and histological assessments of partial signet ring cell carcinoma in a 67-year old male patient. (a) An enhanced CT scan image, showing an enhancing lymph node at the arterial phase with an irregular border in right mesorectum. (b) A high-resolution MR T2WI scan image, showing an irregular edge of a lymph node. (c) An enhanced T1WI (slice thickness 1 mm) scan image, showing a significant high signal intensity of lymph node. (d) A DWIBS scan image, showing a high signal intensity node. (e) Photomicrograph (×40) of HE stained specimen, confirming histologically invasion of the nodal capsule by tumor cells, proliferation of fibrotic tissue, and irregular marginal zone of the node.

**Figure 2:**
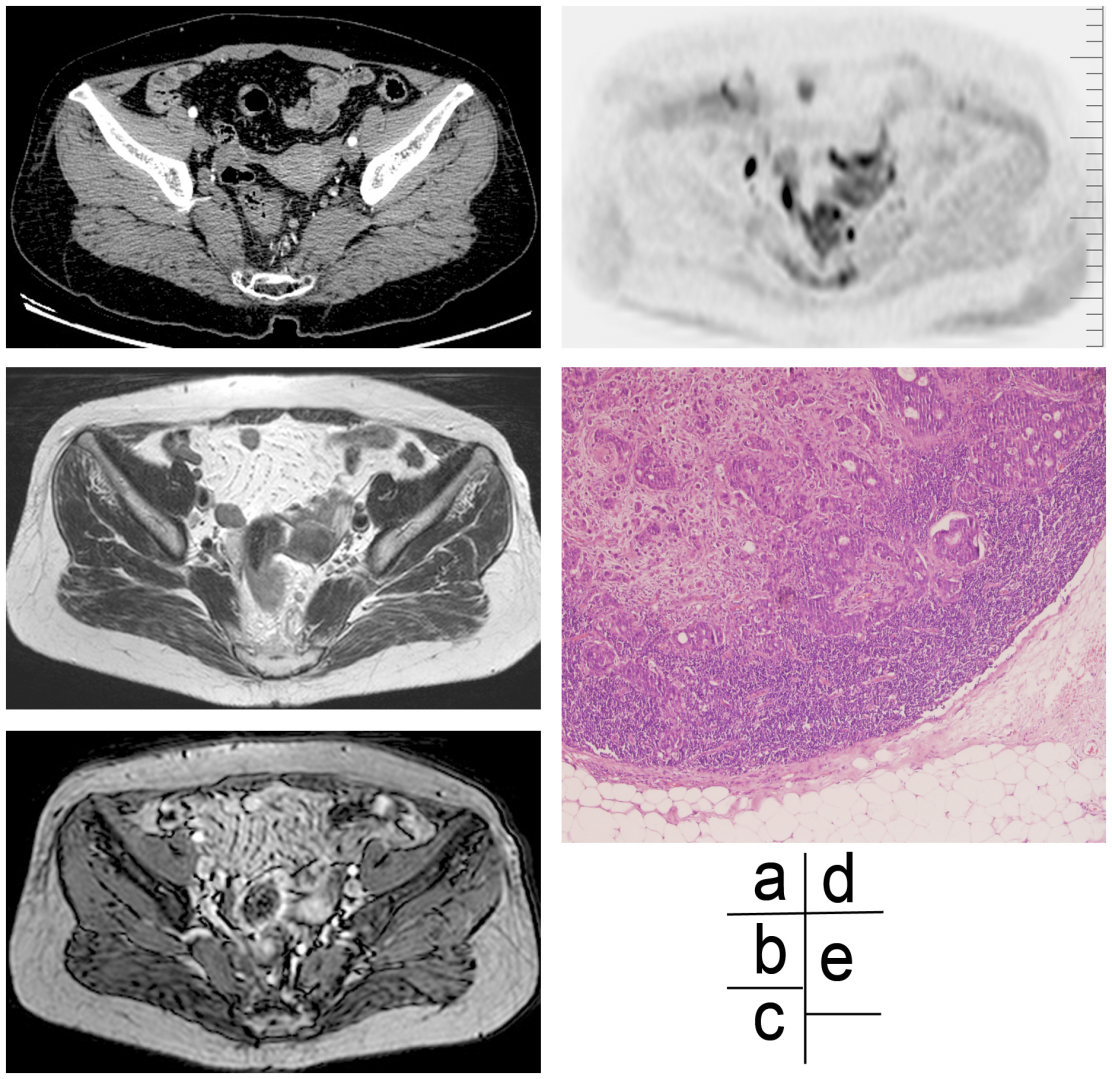
Imaging and histological assessments of partial mucinous adenocarcinoma and signet ring cell carcinoma in a 61-year old female patient. (a) An enhanced CT scan image at the arterial phase, showing an enhancing lymph node with a smooth border in left mesorectum. (b) A high-resolution MR T2WI scan image, showing a smooth-edge of a node. (c) An enhanced T1WI (slice thickness 1 mm) scan image, showing a significant high signal intensity of a lymph node. (d) A DWIBS scan image, showing a high signal intensity of a lymph node. (e) Image of a HE stained specimen (×40), showing the smooth-edge of a lymph node, metastatic tumor tissues in cortex and medulla of lymph node, irregular glandular configuration, and highly heterogeneity cells.

**Figure 3:**
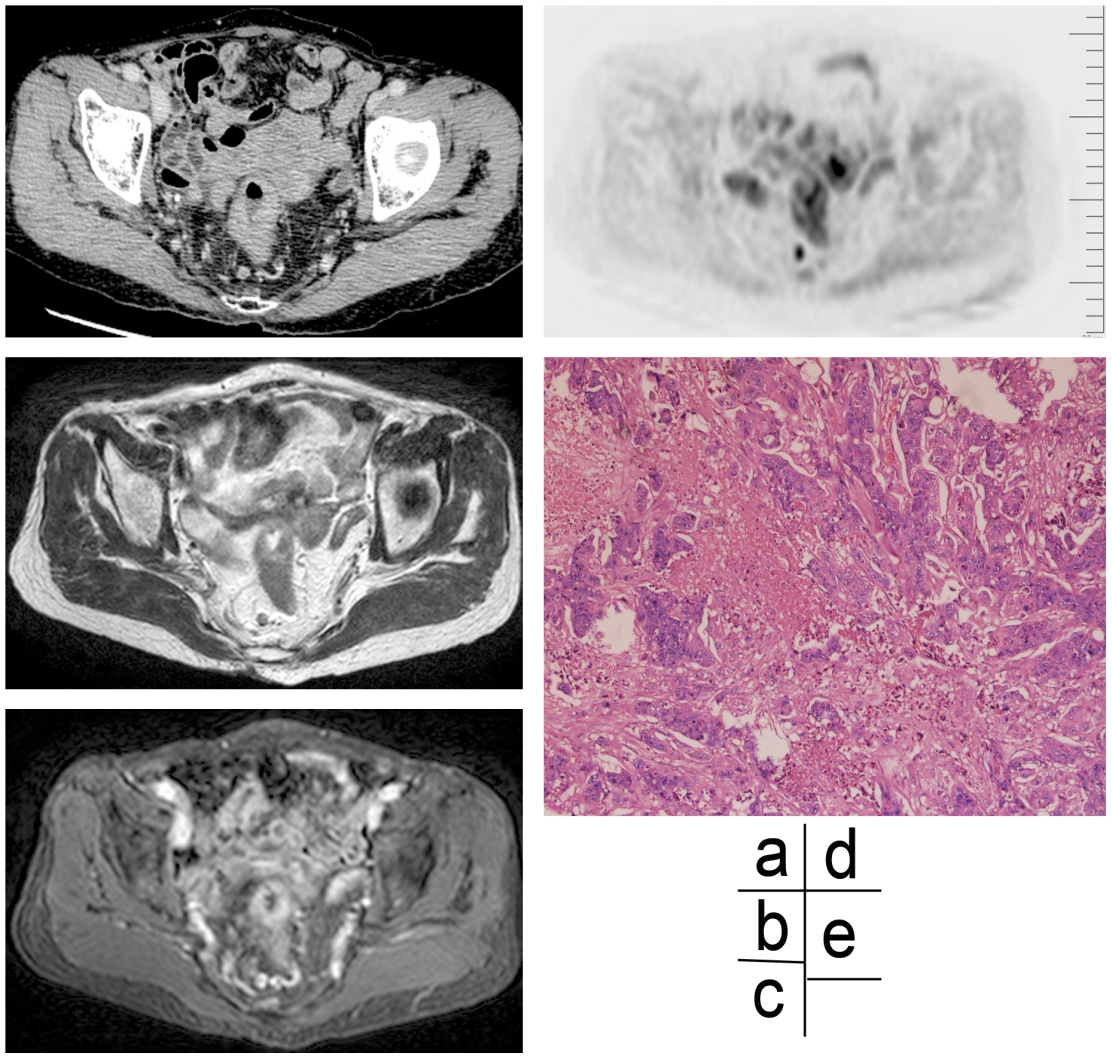
Imaging and histological assessments of rectal carcinoma in a 78-year old female patient. (a) An enhanced CT scan image at the arterial phase, showing a rim-enhancing lymph node with an irregular border in right mesorectum. (b) A high-resolution MR T2WI scan image, showing an appearance of irregular edge in a node. (c) An enhanced T1WI (slice thickness 1 mm) scan image, showing a significant enhancement of the lymph node edge. (d) A DWIBS scan image, showing a high signal intensity node. (e) Photograph (×100) of a HE stained specimen, showing the presence of tumor tissues in various shapes and sizes coagulative necrosis in the lymph node.

As shown in Table 2, preoperative CT and MRI nodal staging data had a fair agreement with pathologic results and the Kappa value (0.331 and 0.348, respectively, P<0.01 for both), and preoperative DWIBS nodal staging data had a relatively lower but still significant agreement with pathologic results and Kappa value (0.174, P<0.05). The accuracy of CT and MRI was 57.7% and 59.6%, respectively, in determining nodal staging in terms of the lymph node border contour, and 57.7% and 61.5%, respectively, in determining nodal status in terms of the lymph node enhancement pattern (Figures 1–3).

**Table 2:** Relationship between preoperative CT, MR, and DWIBS nodal staging and the pathologic results (cases, n = 52).

Imaging methods	Kappa value	P value
Enhanced CT	0.331	P<0.01
MRI	0.348	P<0.01
DWIBS	0.174	P<0.05

P<0.05: statistically different.

## Discussion

It is important to identify patients with nodal involvement because patients with nodal metastasis need radiotherapy or an aggressive lymph node dissection [15]. The present study first demonstrates the comparison of preoperative prediction of nodal involvement in patients with primary rectal carcinoma through three imaging modalities including CT, MR imaging, and DWIBS.

Previous report had shown that nodal disease was associated with a higher rate of local recurrence [25]. Depth or extent of tumor invasion in the rectal wall and lymph node involvement have been shown to be a major prognostic factor in determining the risk of recurrence in rectal cancer; for example, a Dutch trial demonstrated that lymph node status is an important prognostic factor independent of the width of the resection margin [26]. Recently, several studies have reported the beneficial effects of neoadjuvant chemoradiotherapy in terms of a reduced local recurrence rate, and neoadjuvant chemoradiotherapy before rectal resection with TME has been widely accepted as the standard treatment for locally advanced rectal cancer [27]. Therefore, preoperative detection of lymph node metastases in patients with rectal cancer will play a crucial role in treatment planning, in avoidance of local lymph node recurrence and in prognosis [28].

In our study, 14 of these 52 patients had T2 stage tumor, 35 of these 52 patients had T3 stage tumor and the remaining three had T1 stage tumor. This research mainly focuses on primary rectal carcinoma patients (stage T2 and T3 tumors) with absence of tumor invasion into pelvic structures [13]. These patients underwent total mesorectal excision (TME) during two weeks after radiological examinations. Mesorectal, pelvic and retroperitoneal lymph nodes were removed through TME surgery. Patients with stage T4 tumors generally underwent preoperative radiotherapy and/or chemotherapy in order to reduce the tumor staging.

Preoperative nodal staging with imaging modalities (CT, MRI and DWIBS) was compared with the final histological findings. Then, we found the nodal staging agreement between imaging and pathology was fairly strong for CT and MRI (Kappa value = 0.331 and 0.348, P<0.01) but was relatively weaker for DWIBS (Kappa value = 0.174, P<0.05). The sensitivity for CT (78.3%) was higher than that for MR (56.5%), since the lymph node size was not the only assessment criteria of nodal staging. In contrast, the positive predictive value (64.3%) and accuracy (57.7%) for CT were both lower than for MRI (72.2%, 63.5%), possibly due to the overestimation of lymph nodes with an irregular border and a heterogeneous pattern of enhancement, which was consistent with the data reported in the previous literature [12], [15], [23], [29].

The conventional evaluation of lymph node metastasis has been based mainly on nodal size, although there is little consistency in the size used to discriminate between benign and malignant lymph nodes. Normal lymph nodes, reactive lymph nodes, and metastatic lymph nodes in patients with rectal carcinoma may frequently overlap. Compared with other pelvic tumors, lymph node micrometastasis is easily detected in normal-sized lymph node of primary rectal carcinoma patients [29]. However, a recent pathologic study demonstrated that the optimal cut-off value of the long-axis diameter was 8.4 mm with an accuracy of 71.9% [30]. Therefore, prediction of nodal metastases based on CT and MRI assessment of the lymph node size is of important clinical significance in patients with rectal cancer.

In this study, N category staging was determined by the size over 4 mm, border characteristics and enhancement patterns of the lymph node. Our results showed that the accuracy of nodal staging with CT and MR was slightly lower than that of the overall N category staging when differentiating malignant lymph nodes from benign ones according to the border characteristics or signal intensity of nodal morphology. When nodes with the long-axis of 4 mm and over were analyzed, the accuracy of the morphological N staging with MR was slightly higher than that with CT. This was consistent with findings from a previous study [14].

Reactive lymph and metastatic lymph nodes is hard to distinguish when the lymph node counter was used as the criteria, however, Brown et al. [12] reported that if a node was defined as suspicious because of an irregular border or mixed signal intensity, a superior accuracy was obtained and resulted in a sensitivity of 51 (85%) of 60 and a specificity of 216 (97%) of 221 (95%). Reports on preoperative assessment of lymph node status in patients with rectal cancer with enhanced MRI or CT are relatively rare. Unequally enhanced low signal intensity represents mainly a tumor with necrosis or extracellular slime substance. We found that the accuracy for enhanced MR (61.5%) in determining nodal staging was relatively higher than that for CT (57.7%), but still significantly lower than that for convention MR (87.9%) in our study. A recent study has shown that computer-aided quantitative analysis can improve the prediction of node status in rectal cancer [31], it provide a new way for CT to be applied in the determination nodal staging.

Recently, diffusion-weighted magnetic resonance imaging (DWI) has become a useful adjunct for assessing tumors with magnetic resonance imaging (MRI). DWI involves the acquisition of a magnetic resonance signal related to random thermal motion (Brownian motion) which can be reduced by structural barriers, such as cell membranes and collagen [21]. In this report, we assessed nodal staging by lymph node size combined with signal intensity of DWI; we found that the sensitivity and negative predictive value for DWIBS were both 100%. However, the accuracy for DWIBS was only 40.4%, which indicates that using these measures will benefit the detection of lymph nodes in sensitivity, specificity, and negative predictive value. The application of DWI has been extended to oncologic imaging throughout the body in recent years [23], [32], [33]. The use of DWI complementary to conventional MRI methods has led to improvements in the detection and characterization of tumors, treatment response monitoring, and detection of recurrence in cancer patients. It can be expected that with the further development of MRI, DWI will play a more important role in the assessment of lymph node metastasis. The high sensitivity of DWIBS could improve the analysis efficiency of lymph node involvement in rectal cancer, but it is currently unlikely to be useful in clinical practice due to its low accuracy.

This study was associated with some limitations. Firstly, selection bias may occur because some patients with T1 tumors were excluded due to the lack of CT and MR examinations before operation. Secondly, in our study, all patients underwent TME within two weeks after CT and MR examinations, and therefore the lag between surgical operation and radiological examinations may cause an underestimation of theoretically possible on-going lymph node metastasis. Finally, though established on the basis of surgery and histopathological findings, the reference standard we adopted could be still difficult to fit small lymph nodes.

In conclusion, MRI is relatively more accurate than CT in predicting nodal involvement in patients with primary rectal carcinoma in the absence of tumor cell invasion to pelvic issues. Then, we recommend that in clinical practice patients with rectal cancer should undergo MR examination first; followed by DWIBS examination of size, border characteristics, and enhancement patterns of the lymph node, whenever nodal involvement is suspected. As a new imaging technology, DWIBS can provide nodal staging and differential diagnosis with high sensitivity and negative predictive value in primary rectal cancer patients. It is particularly useful in differentiating small malignant from benign lymph nodes. Nevertheless, our observations need to be validated in further studies with a larger sample size.
